# The predictors of glucose screening: the contribution of risk perception

**DOI:** 10.1186/1471-2296-15-108

**Published:** 2014-06-04

**Authors:** Pilar Lavielle, Niels Wacher

**Affiliations:** 1 Clinical Epidemiology Research Unit, Hospital de Especialidades, Centro Medico Nacional Siglo XXI, Instituto Mexicano del Seguro Social, Cuauhtémoc 330, Colonia Doctores, 06720, Cuauhtémoc, Distrito Federal, México City, México; 2 Hospital General de Mexico, Federal Health Ministry, Mexico City, Mexico

**Keywords:** Diabetes, Risk perception, Glucose screening

## Abstract

**Background:**

The prevention of type 2 diabetes is a challenge for health institutions. Periodic blood glucose screening in subjects at risk for developing diabetes may be necessary to implement preventive measures in patients prior to the manifestation of the disease and to efficiently diagnose diabetes. Not only medical aspects, but also psychological and social factors, such as the perception of risk (the individuals’ judgment of the likelihood of experiencing an adverse event) influence healthy or preventive behaviors. It is still unknown if risk perception can have an effect on health behaviors aimed at reducing the risk of diabetes (glucose screening). The objective of study was to identify factors that influence glucose screening frequency.

**Methods:**

Eight hundred randomized interviews, which were stratified by socioeconomic level, were performed in Mexico City. We evaluated the perception of risk of developing diabetes, family history, health status and socioeconomic variables and their association with glucose screening frequency.

**Results:**

Of the study participants, 55.6% had not had their glucose levels measured in the last year, whereas 32.8% of the subjects reported having monitored their glucose levels one to three times per year and 11.5% had their levels monitored four or more times per year. Risk perception was significantly associated with the frequency of blood glucose screening. Having a first-degree relative with diabetes, being older than 45 years and belonging to a middle socioeconomic level increased the probability of subjects seeing a doctor for glucose screening.

**Conclusions:**

Glucose screening is a complex behavior that involves the subjects’ perception of threat, defined as feeling vulnerable to the development of diabetes, which is determined by the subject’s environment and his previous experience with diabetes.

## Background

The prevention of type 2 diabetes is a challenge for health institutions. Periodic blood glucose screening in subjects at risk for developing diabetes may be necessary to implement preventive measures in patients prior to the manifestation of the disease and to efficiently diagnose diabetes [1,2].

There is evidence indicating that not only medical aspects, but also psychological and social factors, influence healthy or preventive behaviors. These include the perception of risk, defined as the individuals’ judgment of the likelihood of experiencing an adverse event [3,4]. Therefore, traditionally, in prevention programs, communication about risk has been regarded as one of the key preventive strategies. It was assumed that by receiving information, people would modify their behavior to reduce their risk [5]. Specifically, this meant that at the moment of making a decision concerning a specific behavior, individuals would adhere to the basic principles of a rational choice, including understanding the received information, weighing its importance and making a decision that would optimize the expected value of the outcome. However, evidence has demonstrated that people often fail to weigh the information; rather, their decision model is often intuitive. In such cases, individuals evaluate the consequences differently, according to their own values and priorities [4]. Therefore, even though knowing about risks is a first step for people to adopt and sustain behavioral changes; other factors are involved in this process.

In this sense, the relationship between knowing the risk factors for developing a disease and the adoption of preventive behaviors must not be regarded as causal. People may know “rationally” that they are exposed to a risk factor, but unless they conceive that some personal aspect is under threat, subjects often do not perceive themselves as being vulnerable [6]. As such, perceived risk is an essential component of the majority of models of health behavior, such as the Health Belief Model, the Health Promotion Model and the Psychometric Paradigm [7,8].

It is still unknown if risk perception can influence on health behaviors aimed at reducing the risk of diabetes [9]. Therefore, understanding the relationship between risk perception and preventive health behaviors in diabetes is an important issue for many reasons. Specifically, studies about risk perception and behavioral change are limited to specific behaviors. Additionally, risk perception is a multidimensional concept that includes the evaluation of the severity and probability of the event. Finally, the relationship between risk perception and health behaviors can be influenced by contextual factors.

Hence, the purpose of this study was to assess the role that risk perception can play and to determine: a) risk perception influences an individual’s behavior for preventing type 2 diabetes, specifically glucose screening; and b) the contextual variables, such as age, sex, socioeconomic status and family history of diabetes, independently influence an individual’s behavior regarding the prevention of type 2 diabetes.

## Methods

### Subjects

After receiving Research and Ethics Committee approval (Comite Local de Investigacicion del Hospital de Especialidades “Dr. Bernardo Sepúlveda G.” reference number 162), we conducted a population-based survey designed to be representative of two different middle and low economic strata from Mexico City. The information about economic strata, which depends on indicators of infrastructure, quality and equipment of households, health, education and employment, was obtained from The National Institute of Statistics, Geography and Informatics, a government agency responsible for population census.

To cover the estimated sample size, we used a probability-clustered sample from six basic census geographic areas (census tracts). Within the neighborhoods, the blocks were subdivided so that each neighborhood would provide approximately 425 homes. Subjects without diabetes aged ≥18 years old were interviewed in their homes. In order to be included in the sample, all subjects were asked to give verbal informed consent. The response rate for the household interviews was 90%.

### Data collection instruments

We measured the strength of subject’s beliefs about risk perception. The instrument included two questions about their perception of the risk of developing diabetes, including likelihood and severity. These questions were developed based on factors that emerged in previous risk perception research [10,11]. One question assessed the belief that one is vulnerable to being affected by a particular health problem (“How likely are you to get diabetes?”), and the question on “severity” asked if the subjects considered diabetes a serious disease (“How serious is getting diabetes?”). A visual analogue scale was used that ranged from 1 to 10 points, representing not at all likely, serious to very likely and serious. The reliability for these questions was measured by correlation coefficients of a 5-day test-retest; the correlations observed ranged from 0.52 to 0.94 (the p values ranged from 0.001 to 0.031) [12]. The perceived likelihood and severity were summarized into one combined variable that, according to factor analysis, was integrated into one factor that explained 62.7% of the variance. This variable was then dichotomized into two groups representing good and poor risk perception for analysis.

The participants were asked about some beliefs about diabetes. The instrument included the three following questions about diabetes: knowledge (“How much do you know about diabetes?”), responsibility (“Are you responsible for getting diabetes?”) and concern (“Are you concerned about getting diabetes?”).

We assessed risk reduction behavior by asking all participants whether they had visited a physician in the past 12 months for a blood glucose test (“How often did you go to the medical clinic in order to have your blood glucose measured?”). Other risk factors, such as having a first-degree relative with diabetes, were also considered. Finally, for health status, we used self-assessment, which has been proven to be a reliable measure associated with the actual general state of health and mortality [13]. For its evaluation, a question was included on how the subject perceived his/her health status. This question used a 10-point visual analogue scale in which 1 corresponded to a very poor health status and 10 to an excellent health status. The scores obtained were converted into three categories, according to percentiles, for their analysis.

### Statistical analysis

The association between risk perception and preventive behavior, specifically, attending a medical clinic for glucose measurement, was estimated using the Chi-square test. We also used single logistic regression to model the likelihood of adopting glucose screening (dependent variable) among adults without diabetes according to risk perception, age, gender, socioeconomic status and the presence of a family history of diabetes (independent variables). The predicted margins and 95% confidence intervals (CIs) were estimated, and the level of statistical significance was set at 0.05.

## Results

Eight hundred subjects were interviewed, with 400 belonging to a low socioeconomic level and 400 to a middle level. A total of 15 subjects were excluded because their questionnaires were incomplete. More than half of the total sample population was women (54.3%). The mean participant age was 41.3 ± 16.4 (range 18–92) years. The majority (58.7%) reported that they were married. A large proportion of the subjects did not have a college education and predominantly held technical and non-qualified jobs, such as tradesmen or women, factory workers, carpenters and construction workers (Table 1).

**Table 1:** Sample demographic characteristics

	**%**	**n**
**Sex**		
Male	45.7	359
Female	54.3	426
**Age (years)**		
< 45	61.1	480
≥ 45	38.9	305
**Marital status**		
Single	30.3	238
Married	58.7	461
Divorced	5.9	46
Widow	5.1	40
**Education**		
Basic (≤ 9 years)	39.7	312
Middle (12 years)	28.7	225
High (≥13 years)	31.6	248
**Occupation**		
Non-qualified	35.4	278
Technician	24.3	191
Home	19.2	151
Student	8.4	66
Professional	8.2	64
Retired	4.5	35

With regard to preventive behavior, 55.6% of the total sample population had not had their glucose levels measured in the last year, whereas 32.8% of the subjects reported having their blood glucose levels measured between one and three times per year and 11.5% had their levels measured four or more times per year. Additionally, 49.6% mentioned they had a family member with diabetes. The health status of the subjects was rated as low, with a mean score of 7.61 ± 1.76. The participant’s mean score for combined variables of risk perception, including both the likelihood and severity perception of diabetes, was 6.77 ± 2.5.

Of all of the study participants, 38.7% considered themselves responsible for developing diabetes, while 70.3% indicated having high concern of developing diabetes. With regard to level of diabetes knowledge, 90.3% rated themselves as having high and moderate levels of knowledge.

When the relationship between preventive behaviors and socio-demographic, clinical and risk perception variables was explored, most of these variables were found to have a considerable impact on the preventive behavior under study. The variables that had no relationship with preventive behavior were sex and health status (see Table 2).

**Table 2:** Preventive behavior and their relationship with socio-demographic, clinical and risk perception variables

	**Glucose measured n**	**%**
**Sex**		
Male	150/359	41.8
Female	196/426	46.0
**Age**		
< 45	165/480	34.4^a^
≥ 45	181/305	59.3
**Socioeconomic status**		
Low	133/389	34.2^a^
Middle	213/396	53.8
**Family history**		
No	152/396	38.4^a^
Yes	194/389	49.9
**Health status**		
Bad	77/172	44.8
Regular	168/379	44.3
Good	101/234	43.2
**Risk perception**		
No	163/424	38.4^a^
Yes	183/361	50.7

^a^p ≤ 0.05.

All variables that were identified by bivariate analysis to have statistical significance in predicting glucose screening were included in a single logistic regression model. Table 3 shows the variables that were independently associated with glucose measurement, specifically, age (odds ratio [OR] 0.34 95% CI, 0.25–0.47) and risk perception of diabetes (OR 0.63 95% CI, 0.46–0.86). Finally, contextual factors, such as having a first-degree relative with diabetes (OR 0.57 95% CI, 0.42–0.79) and belonging to a middle socioeconomic level (OR 0.45 95% CI, 0.33–0.61), increased the proportion of subjects seeing a doctor to have their glucose levels measured.

**Table 3:** Variables independently associated with preventive behaviors results of logistic regression analysis

**Variables**	**OR (95% CI)**	**p**
Age (>45 years)	0.34 (0.25–0.47)	0.00
Socioeconomic status (middle)	0.45 (0.33–0.61)	0.00
Family history	0.57 (0.42–0.79)	0.01
Risk perception	0.63 (0.46–0.86)	0.04

## Discussion

This study about glucose screening demonstrated that a high proportion of participants had never performed this preventive behavior. Attending a medical clinic to determine one’s glucose level was associated with risk perception, age (>45 years), history of diabetes and socioeconomic status (middle). This finding is relevant because it has been reported that at least one-third of people do not attend their glucose screening appointments [14]. While general measures, such as exercising and avoiding becoming overweight, may be adequate for the general population to prevent diabetes development, more strict measures may be needed for those in higher risk categories, such as pre-diabetic patients. Additionally, glucose screening may be needed to identify such higher risk individuals.

Glucose screening was significantly and independently related to the individuals’ perception of the likelihood and severity of developing diabetes. The subjects in our study were less willing to adopt preventive behaviors because they did not perceive the condition as threatening, as they considered themselves to be hardly vulnerable to diabetes development. This may result from the tendency to use defensive cognitive strategies, such as risk minimization, to avoid feelings of distress. As such, this cognitive optimism bias may lead to an erroneous evaluation of their probability for suffering any damage to their health [15-17]. Additionally, this underestimation of risk could depend on specific characteristics of diabetes, such as consequences that may not be observable or that do not occur immediately [18-21].

In agreement with other studies, our data confirms that, in addition to risk perception, contextual factors also influence a subject’s motivation for attending glucose screening tests. We found that previous experience with the condition, such as having a family history of diabetes, increased the proportion of subjects measuring their glucose levels [22]. Furthermore, similar to previous reports, we found that older subjects measured their glucose more often and adhered more to their physicians’ recommendations [23].

Previous studies have demonstrated that belonging to a low socioeconomic group affects an individual’s health behaviors [24]; in our study, we found that only 44.6% of participants went to a medical clinic to measure their glucose levels. There are some possible explanations for this low proportion. Belonging to a low socioeconomic group affects the capacity of subjects to protect their health because socioeconomic level, as a complex construct, entails differences in education, resources and “cultural competency” [25]. Accordingly, socioeconomic level has psycho-social effects on an individual, such as increasing the likelihood of experiencing feelings of fatalism, decreasing expectations regarding the future and reducing motivation toward learning new coping strategies [26-28].

This study is limited because only one health behavior, glucose screening, was examined. More complex behavioral changes for lifestyle modification may be dependent on additional variables, such as the perception of self-efficacy, that were not explored in this study. Nevertheless, within these limits, our findings are still relevant because only one other study has studied a population-based sample.

## Conclusions

In summary, periodic glucose screening is a complex behavior that involves the subjects’ perception of threat, defined as feeling vulnerable to the development of diabetes, which is determined by the subject’s environment and his previous experience with diabetes.

## Competing interests

The authors declare that they have no competing interests.

## Authors' contributions

PL conceived of the study, performed the statistical analysis, and contributed to the writing and editing of the manuscript. NW contributed to the writing and editing of the manuscript. Both authors read and approved the final manuscript.

## Pre-publication history

The pre-publication history for this paper can be accessed here:

http://www.biomedcentral.com/1471-2296/15/108/prepub
